# Decision-making preceding induced abortion: a qualitative study of women’s experiences in Kisumu, Kenya

**DOI:** 10.1186/s12978-018-0612-6

**Published:** 2018-10-03

**Authors:** Ulrika Rehnström Loi, Matilda Lindgren, Elisabeth Faxelid, Monica Oguttu, Marie Klingberg-Allvin

**Affiliations:** 1 Department of Public Health Sciences/IHCAR, Karolinska Institutet, SE-171 77 Stockholm, Sweden; 2 College of Health Sciences, School of Nursing Sciences, University of Nairobi, Nairobi, Kenya; 3 Kisumu Medical Education Trust (KMET), Kisumu, Kenya; 4 Department of Women’s and Children’s Health, Karolinska Institutet, SE-171 77 Stockholm, Sweden; 5 School of Education, Health and Social Studies, Dalarna University, Högskolegatan 2, 791 31 Falun, Sweden

**Keywords:** Abortion, Decision-making, Qualitative methodology, In-depth interviews, Kenya

## Abstract

**Background:**

Unwanted pregnancies and unsafe abortions are prevalent in regions where women and adolescent girls have unmet contraceptive needs. Globally, about 25 million unsafe abortions take place every year. In countries with restrictive abortion laws, safe abortion care is not always accessible. In Kenya, the high unwanted pregnancy rate resulting in unsafe abortions is a serious public health issue. Gaps exist in knowledge regarding women’s decision-making processes in relation to induced abortions in Kenya. Decision-making is a fundamental factor for consideration when planning and implementing contraceptive services. This study explored decision-making processes preceding induced abortion among women with unwanted pregnancy in Kisumu, Kenya.

**Methods:**

Individual face-to-face in-depth interviews were conducted with nine women aged 19–32 years old. Women who had experienced induced abortion were recruited after receiving post-abortion care at the Jaramogi Oginga Odinga Teaching and Referral Hospital (JOOTRH) or Kisumu East District Hospital (KDH) in Kisumu, Kenya. In total, 15 in-depth interviews using open-ended questions were conducted. All interviews were tape-recorded, transcribed and coded manually using inductive content analysis.

**Results:**

Respondents described their own experiences regarding decision-making preceding induced abortion. This study shows that the main reasons for induced abortion were socio-economic stress and a lack of support from the male partner. In addition, deviance from family expectations and gender-based norms highly influenced the decision to have an abortion among the interviewed women. The principal decision maker was often the male partner who pressed for the termination of the pregnancy indirectly by declining his financial or social responsibilities or directly by demanding termination. In some cases, the male partner controlled decision-making by arranging an unsafe abortion without the woman’s consent. Strategic choices regarding whom to confide in were employed as protection against abortion stigma. This contributed to a culture of silence around abortion and unwanted pregnancy, a factor that made women more vulnerable to complications.

**Conclusions:**

The findings suggest that financial, social and gender-based dependencies influence women’s agency and perceived options in decision-making regarding abortion.

## Plain English summary

Unwanted pregnancies and pregnancy termination are common in countries where women who want to prevent or delay childbearing have limited access to contraceptives. Around 25 million unsafe abortions take place worldwide each year. Recent evidence shows that nearly half a million induced abortions take place in Kenya every year. In this study, we used in-depth interviews to explore the decision-making processes preceding induced abortion among women with unwanted pregnancies in Kisumu, Kenya.

This study shows that the interviewed women decided to terminate their pregnancies for the following reasons: poverty, poor timing of the pregnancy and absence of support from male partners. The main decision maker was usually the male partner who pressed for the termination of the pregnancy indirectly by declining his financial or social responsibilities or directly by forcing his partner to terminate the pregnancy. In some cases, the male partner arranged an unsafe abortion without the woman’s knowledge or consent. Participants were affected by social stigma and carefully selected whom to talk to about the abortion. This strategy was used as protection against humiliation and shame. This contributed to a culture of silence around abortion and unwanted pregnancy, a factor that made women vulnerable to complications.

## Background

Where women and adolescent girls have unmet contraceptive needs, unwanted pregnancies and unsafe abortions are common. About 25 million unsafe abortions (45% of all induced abortions) occur globally, most of them (97%) in low resource settings [1]. Despite the availability of safe and effective interventions, unsafe abortions still contribute to maternal morbidity and mortality [2]. The majority of maternal deaths due to unsafe abortions occur in low-income settings where women experience low social status combined with legal and social restrictions to sexual and reproductive rights [3]. Women tend to opt for unsafe abortions where safe abortion services are not acceptable, accessible or affordable [4]. The number of unsafe abortions tends to be higher among poor women because women with strong social or economic resources are more likely to access safe abortions, regardless of the legal context [5].

The World Health Organization (WHO) defines unsafe abortion as “*the termination of an unwanted pregnancy by persons lacking the necessary skills, or in an environment lacking minimal medical standards, or both”* [6], while also emphasising the impact of the social and legal context on abortion safety [7]. A recent study showed the disparity in abortion safety between low- and high-resource settings, indicating that in high-resource settings almost all abortions were safe, while only one in four abortions in Africa were safe [1].

The 2030 Agenda for Sustainable Development renewed the commitments by 193 Member States of the United Nations to reduce global maternal mortality through universal access to sexual and reproductive health (SRH) services, education and information. Moreover, sexual and reproductive health and rights (SRHR), ensuring the ability to make decisions about one’s contraceptive and own health, is core to the post-2015 goals because of its remarkable potential to contribute to sustainable development [8].

Contraceptives allow women/couples to decide if and when to become pregnant. Modern contraceptives play an important role in reducing maternal deaths by preventing unwanted pregnancies and prolonging birth intervals [9]. Contraceptives are, however, underutilised in many low-resource settings [10, 11], largely as a result of limited availability of a range of contraceptive methods, including to modern long-acting reversible contraceptive methods [11], and social stigma surrounding young women’s contraceptive use [12].

Women’s decision-making preceding an induced abortion is influenced by factors at different levels [13, 14]. Individual-level factors include marital status, education level, economic independency and whether the woman was a victim of rape or incest [15]. Interpersonal factors such as parental and partner support have also been found to influence decision-making [15], as have societal determinants like religion and social stigma and norms [13]. Relevant organisational factors include access to sexuality education [15] and the availability of facilities providing abortion services [14].

### The Kenyan context

The majority of the population in Kenya is Christian (83%), with 48% identifying as Protestant and 24% as Roman Catholic [16]. Kenyan women are economically dependent on men, and Kenyan cultures are largely patriarchal [17]. Marriage occurs comparatively early; among women aged 25–49 the median age at first marriage was 20.2 years. About 53% of married women of reproductive age use a modern contraceptive method. Among married women aged 15–49 years, 18% have unmet contraceptive need, which contributes to a high total fertility rate (3.9 births per woman) [18].

A recent national study estimated that about 464,000 induced abortions occur in Kenya annually, with a national abortion rate of 48 abortions per 1000 women of reproductive age (15–49 years) [19]. This figure is above the rate for all of sub-Saharan Africa (SSA), which is 31 abortions per 1000 women of reproductive age [20]. It is estimated that the induced abortion rate in Kenya is highest in the Rift Valley region and the combined Nyanza and Western regions [19].

Until 2010, abortion was only legally allowed to save the life of a pregnant woman. However, in 2010 a revised constitution was adopted permiting abortion when “*in the opinion of a trained health professional, there is need for emergency treatment, or the life or health of the mother is in danger, or if permitted by any other written law*” [21]. Thus far, the implementation of the constitution has been slow, and both knowledge and practice may differ throughout the country. A lack of transparency and clarity with regard to the circumstances in which abortion is legal contributes to Kenya’s high maternal mortality ratio (MMR) [22]. The MMR in Kenya has remained almost constant since 1990. According to the 2014 Kenya Demographic Health Survey, the MMR is 362 maternal deaths per 100,000 live births, and unsafe abortion is a major contributor [18]. Due to restrictive abortion legislation in Kenya [21], limited access to quality healthcare and stigma, most abortions occur outside authorised health care facilities and are classified as therefore considered unsafe [23].

Kenya is an important location to study women’s decision-making preceding induced abortion given its high MMR, changing legal framework, social stigma surrounding unplanned pregnancies and the socioeconomic status of the majority of women in the country.

Nyanza province, in which Kisumu is the principal city, has one of the highest MMRs in Kenya [24], and the total fertility rate for this province is 4.3 children per woman, the fourth highest in the country [18].

### Aim of the study

The aim of this study was to explore decision-making preceding induced abortion among women with unwanted pregnancies in Kisumu, western Kenya.

## Methods

### Study setting

The study was conducted at the Jaramogi Oginga Odinga Teaching and Referral Hospital (JOOTRH) and Kisumu East District Hospital (KDH) in Kisumu, western Kenya. Kisumu Medical and Education Trust (KMET), a non-governmental organisation, supported the collaboration with these two public hospitals in Kisumu. At the time of the study, the two facilities treated approximately 80 women per month for abortion-related complications.

### Research team and reflexivity

The authors recognise the significance of reflexivity and transparency regarding researcher subjectivity in qualitative research. The research team consisted of five female researchers. The first author (URL) had prior relevant experience from an MSc in Public Health and as a PhD student in the researched subject. The second author (ML) is a social scientist (MSc student) with an interest in women’s SRH. The third author (EF) is a professor in Reproductive and Perinatal Health Care with broad experience conducting quantitative and qualitative research in Kenya and other low-income countries. The fourth author (MO) is the Executive Director of KMET with vast SRH experience in the region. The final author (MKA) is a professor with a PhD in International Health who has extensive experience conducting research in low-resource settings using both quantitative and qualitative methods.

Conducting and transcribing the interviews was physically and emotionally exhausting. During data collection and interview transcription the researchers (ML and URL) had daily contact and discussed their personal experiences. The deep emotional experience of conducting these interviews allowed them to empathise with participants and was used during analysis.

### Study design, sample selection and data collection

In total, 15 individual, in-depth interviews (IDIs) were conducted with nine women aged 19–32 years old. Follow-up interviews were conducted with six of the women. Purposive sampling was used to select women seeking care for abortion-related complications. The following inclusion criteria were used: 1) women over 18 years of age 2) who experienced an induced abortion, 3) received post-abortion care (PAC) at JOOTRH or KDH and 4) were willing to be interviewed.

Midwives at the two public hospitals in Kisumu identified possible interviewees between 1 January 2014 to 31 May 2014 by asking PAC-seeking women if they had tried to induce the abortion. All women who met the inclusion criteria and were asked to participate agreed to be interviewed. The respondents were informed about the study’s aim and were assured of their confidentiality. Seven respondents were recruited from JOOTRH and two from KDH. Six women were interviewed face-to-face 7–10 days after receiving PAC, two were interviewed at the time of a three-month follow-up and one woman was approached while she was still admitted at the ward. In addition, a repeated interview was offered to all respondents approximately 2–5 weeks after the initial interview. Five of the respondents were interviewed face-to-face a second time, while one respondent was interviewed over the phone due to distance. Three respondents declined the request for a repeat interview. The reason for conducting follow-up interviews was to further enhance understanding and enrich the material as trust and affinity were built between researcher and informant.

The women were interviewed between February and April 2014 at JOOTRH and KDH by one of the authors (ML) who has a master’s degree in Gender Studies and conducted previous studies in Cultural Anthropology. She was trained in qualitative methodologies and at the time of the study was a postgraduate student in Global Health. The fact that the interviewer was not a clinician and a non-Kenyan might have encouraged respondents to speak to her more openly about a sensitive subject. During one interview the researcher used an interpreter to translate from Lou to English. The translator was an assistant from KMET. During the other interviews, the researcher was the only person in the room with the respondent. The interviews lasted on average 45 min.

A semi-structured interview schedule, using open-ended questions and suggestions for probing, was developed by the research team. The schedule was pilot tested and modified prior to initial data collection. The questions were framed to study women’s decision-making preceding induced abortion, including the role played by their social networks.

Field notes were written directly after each interview to reflect on initial thoughts and reactions. With the written consent of the respondents, all interviews were tape-recorded and transcribed verbatim, including notations for nonverbal expressions, for analysis by the first and second authors (URL and ML) on an ongoing basis as data collection progressed. The interview with a translator was also transcribed in English. The research team met regularly to review progress and discuss interview techniques. Data collection continued until data saturation was reached [25]. Table 1 presents the characteristics of the respondents.

**Table 1:** Socio-demographic characteristics of respondents (R) at time of abortion

R (*n* = 9)	Age	No. of children	Relationship status	Occupation	Abortion method	Abortion provider	Other details
1	19	0	Partner	Selling vegetables	Surgical	Not sure	Tricked into abortion by her partner
2	20	0	Partner	University student	Medical	Chemist	Tricked into abortion by her partner
3	22	1	No relationship	University student	Medical	Physician at public hospital	Safe abortion
4	22	0	Partner	College student	Surgical	Chemist	Repeat abortion
5	25	0	Engaged	Primary school teacher	Medical	Physician at private clinic	Safe abortion
6	26	2	No relationship	Food preparation at hotel	Medical	Chemist	
7	29	1	No relationship	Unemployed	Medical	Physician at private clinic	HIV+
8	32	4	Married	Primary school teacher	Surgical	Chemist	HIV+ and pregnant with twins
9	32	4	Married	Unemployed	Overdose of malaria drugs	Self-administered	

### Data analysis

The data were analysed by the first and second authors (URL and ML) using inductive content analysis, including open coding, category development and abstraction [25, 26].

**Fig. 1:**
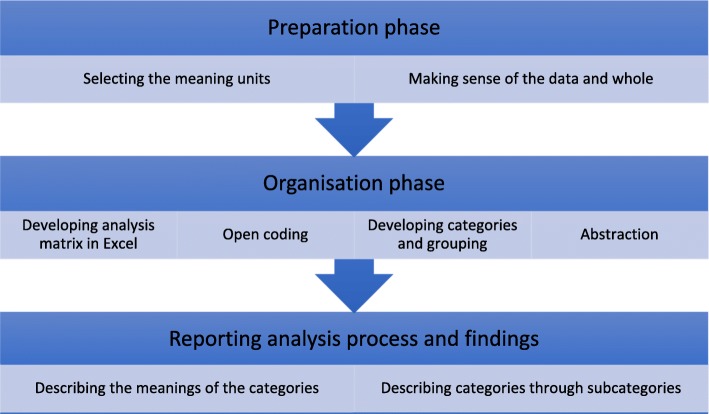
Inductive content analysis process [27]

Inductive content analysis is a qualitative approach used to unconditionally analysing the data [27]. While analysis had already begun during interview transcription, open coding was conducted during the first reading of the transcripts. Thereafter, the transcripts were read through several times and coded manually. Meaning units were identified and transferred to Excel for classification into subcategories, generic categories and main categories. The process of analysis is presented in Fig. 1. Meaning units and categories were discussed and compared amongst all members of the research team in order to further improve the analysis and to maximise rigour [26].

## Results

**Fig. 2:**
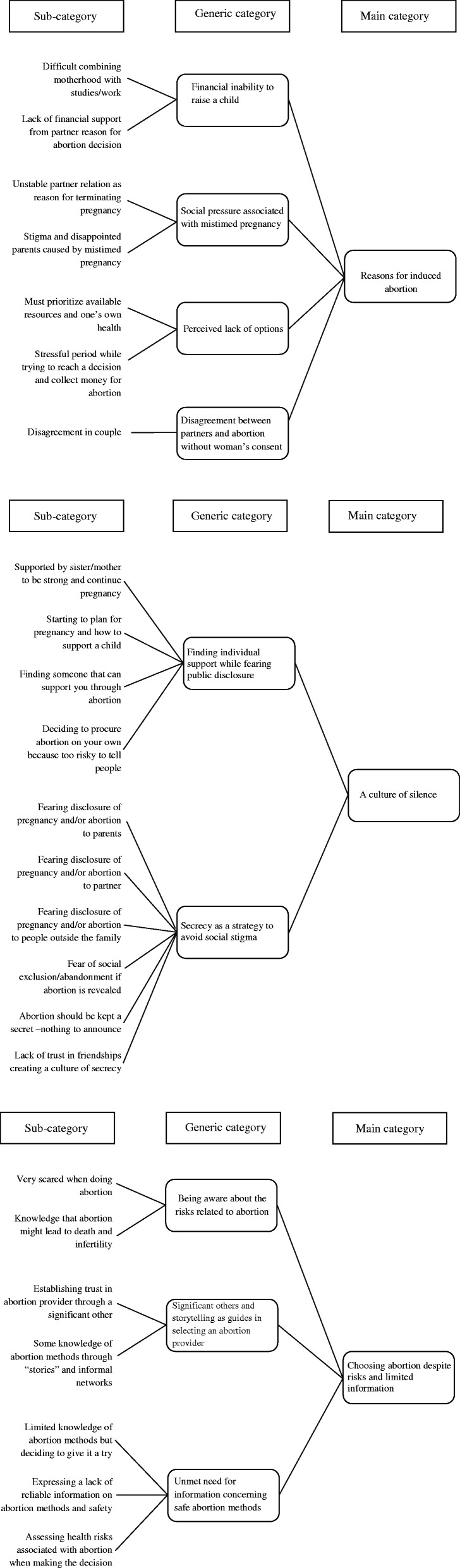
Coding tree describing the abstraction process

Inductive content analysis resulted in three main categories: 1) *Reasons for induced abortion*, 2) *A culture of silence* and 3) *Choosing abortion despite risks and limited information.* The results are presented according to these main categories together with their generic categories and citations from the interviews to clarify the findings. The abstraction process is illustrated in Fig. 2.

### Reasons for induced abortion

The first main category, *Reasons for induced abortion*, is described through four generic categories: 1) *Financial inability to raise a child*, 2) *Social pressure associated with mistimed pregnancy*, 3) *Perceived lack of options* and 4) *Disagreement between partners and abortion without the woman’s consent.*

#### Financial inability to raise a child

All women described their pregnancies as mistimed, unplanned or unwanted at the time of conception. A lack of financial stability or support were described by most women as driving factors for the decision to terminate the pregnancy.

“I was financially unstable to sustain those children.” (Respondent 8)

In some cases, the woman was the main provider of the household, and the pregnancy jeopardised the stability of her income. The pregnancy might diminish her employment opportunities, as an employer could decide to let a woman go once it was evident she was pregnant. Therefore, pregnancy termination provided the respondents with the potential for continued employment and secured economic independence.

“When [the pregnancy] is visible, you will be sacked. And when you are [alone] at home, who will support you? I have to work.” (Respondent 6)

Women who were still students and living with their parents indicated that their parents would not financially support their costs of living and studies as well as the costs of raising an additional child.

The married respondents stressed that they had to prioritise resources and take care of the children they already had. All women with children mentioned the importance of providing an education for them. High school fees were frequently cited. The respondents stated they could not afford to educate an additional child. Several women specifically articulated their partner’s unwillingness to financially support a child as the reason for terminating the pregnancy.

Furthermore, financial constraints were perceived as a barrier to safe abortion. Women frequently cited not being able to afford to pay a professional to perform the abortion.

#### Social pressure associated with mistimed pregnancy

The unmarried respondents were concerned about the risk of negative views from family and community members if they continued the pregnancy at that particular time. Although engaged and employed, some women expected to be criticised and “talked about” by people in the community due to the mistimed pregnancy.

“Because [---] okay, people usually talk; in Kenya people will talk. Where you are staying, there are some people, those people like to gossip, people will definitely talk. [---] They’ll say you are still in your mother’s house [---] They won’t be able to understand…and some will even criticise your relationship.” (Respondent 5)

Others explained that having a child would end a harmonious relationship with their parents. Several young women living with their parents mentioned that they would not be welcome in their parents’ house if they were pregnant.

“[---] she [mother] took me to the training [in hotel management and hospitality], she spend some money there,and then I didn’t tell her [about the pregnancy] because she won’t be happy because maybe she will then think that she had taken me to the training and spent money there, and then I will not be able to go and just sit at home [---] She would kick me out of the house, and maybe she would stop the training [---].” (Respondent 7)

The need for a supportive social network, including a stable partnership, emerged as fundamental to avoid severe conflicts in the decision-making process. Social networks could include actors providing either financial or couched support. Male partners had a significant direct or indirect influence on participants’ decisions to opt for induced abortions. Several women expressed unstable partner relationships as a reason why they had chosen to have an abortion.

“I already have two children, I am everything for these children… I am the mother and the father for these two children, so a third one would be too much problem. I just decided. I have to because that man never convinced me; I was not convinced at all that that man would provide anything.” (Respondent 6)

Single women were afraid to raise a child alone.

“So, I just thought that I have another kid and the father is not contributing with the school fees. Even my mother denied helping me. Yeah, for my kid and me also; so that’s why I decided to do away with the abortion.” (Respondent 7)

The social network was emphasised as central for single mothers. An additional child became an added burden, which could not be placed on family or friends.

#### Perceived lack of options

Some women expressed guilt and distress about lying to their partners and family about the pregnancy. Additionally, women expressed feelings such as heightened shame and self-blame because abortion was perceived as immoral and improper.

“I felt bad because it was like murdering someone, but [---] I felt part of killing the kid because [---] I felt miserable for like a week [---] two weeks.” (Respondent 2)

However, due to their economic, social or health circumstances, the termination of the pregnancy was considered the only available option.

“I didn’t have any option because I just knew that the situation I was in [HIV positive]; I was not able to [---] take care of this baby [---] according to the situation [HIV positive] now I was in.” (Respondent 7)

While some women said that they decided on an abortion immediately upon realising they were pregnant, several respondents described experiencing a lot of stress and ambivalence in trying to decide what to do.

“I was still deciding what to do; I was still doubting. So many things run into my mind until I come with the decision to do [---] to [---] to [---] end the pregnancy. At that time [---] [I] even think I lost [---] [weight] cause [because of the] stress I have [---] having so many stress [---] losing weight cause of the stress.” (Respondent 3)

#### Disagreement between partners and abortion without the woman’s consent

Almost all women expressed some kind of disagreement with their partner in relation to the pregnancy. Some women articulated that they terminated the pregnancy without notifying their partner, fearing the possible consequences of anger, violence and divorce. On the other hand, a few women expressed their intention to abort and were discouraged and warned not to proceed by the partner. Participants articulated that their partners believed abortion was wrong and could cause complications and death. All single respondents decided not to reveal the pregnancy to their ex-partners. Although some of the respondents decided to terminate the pregnancy, others expressed that they were forced or even misled to terminate the pregnancy by their partners. When women were misled, their respective partners attempted to convince them to opt for an abortion. Although the women insisted on keeping the pregnancy, clandestine abortion providers supported the partners to induce abortion without the women’s consent.

“He suggested for the abortion to be done, I told him no. [---] So he insisted, and he insisted. When he saw I’m not participating, he used a trick and told me that if you don’t want then I want to advice you on how to be when you are pregnant and what drugs [to] use. [---] He injected me through a vein and told me it’s to improve the appetite… [---] After injecting that drug I became unconscious. When I returned from my unconsciousness I found myself naked and I was bleeding.” (Respondent 1)

This reveals that unsafe abortion in Kenya sometimes happens without the woman’s consent. During the interview Respondent 1 disclosed she had reported her ex-partner to the police. While the women explained their partners’ motives were based on social embarrassment and financial obligations, how the partners themselves would describe the situation and justify their actions is beyond the scope of this research.

### A culture of silence

The main category *A culture of silence* is described through two generic categories: 1) *Finding individual support while fearing public disclosure* and 2) *Secrecy as a strategy to avoid social stigma*.

#### Finding individual support while fearing public disclosure

As seen under the first main category, several respondents described the time after they discovered they were pregnant as very stressful. While all of them feared public disclosure to some extent, they also expressed the need to tell someone about their condition. In many cases this person was a sister or a friend who had also been through an induced abortion. Most respondents were reluctant to tell their partners because they feared disagreement or abandonment. Among the women who informed their partners about the pregnancy, this confession commonly entailed asking for financial support. A majority of the non-married respondents also acknowledge that they were reluctant to tell their parents and preferred that their partner did not know about the pregnancy.

Respondents tended to keep the pregnancy to themselves for several weeks due to their fear of possible reactions. If the secret was shared, it had to be with a trustworthy person, usually a sister, as a tactic to avoid public disclosure. Furthermore, women feared receiving opposing advice, which could indicate that they had already made the decision to terminate the pregnancy and only sought affirmation.

“I was doubting what to do and on the other hand afraid of sharing with anybody. I believed if I share it with so many people some people will give me other advice, some will give me this; that’s why I ended up sharing with my sister that I’m staying with because I trusted her.” (Respondent 4)

Not infrequently, respondents indicated that they had chosen to state that the pregnancy had ended in a miscarriage instead of an induced abortion. Women made strategic choices regarding whom they confided in. While some women had the support of a sister or a female friend, others assessed the risk of telling someone to be so profound that they decided to keep the secret to themselves, which meant they had no one who could support them.

“I did not ask someone for advice because if you ask one they will start talking about it and everybody will know about it, so I was afraid to talk about it to someone [---] and maybe it will go back to my partner, and I didn’t want that to happen. [---].” (Respondent 8)

Fear of negative consequences and death as a result of the abortion led some women to share their intention to terminate the pregnancy. Women expressed the desire to inform at least one person about the abortion; if there were negative consequences, someone would know where to look for the woman if she were not to return home.

#### Secrecy as a strategy to avoid social stigma

The majority of women expressed fear of rumours, social isolation and judgment if the abortion were revealed. Respondents believed that people in the community would perceive them as “killers”. Additionally, they believed their peers would exclude them and avoid interacting with them. Social stigma and discrimination were expressed as segregation, as well as being perceived as a prostitute, labelled as a murderer, accused of being unfaithful and believed to be a poor candidate for marriage.

**“**In campus if you get pregnant and your boyfriend says I cannot take care of the baby, I’m not the father and stuff, they will start saying you are just like the others [---] maybe you have sex for money? Maybe you don’t know the father of the kid? [---] So, they start calling you names like whore, slut [---] Someone says you are just a whore like anyone else, and after that everyone starts to isolate you [---]” (Respondent 2)

Fear of judgment and losing social respect created a culture of silence, where the harmful nature of rumours and negative responses fostered secrecy and silence surrounding abortion and a mistimed pregnancy. Women became afraid to share their decision to terminate the pregnancy with others, including friends, family and healthcare professionals due to a lack of trust and fear that their confidentiality would not be maintained. Induced abortions were secrets kept to avoid negative reactions.

Religious values and beliefs were apparent in all the interviews. Almost all women mentioned that abortion was a sin and not accepted by their church. Therefore, it was critical that other church members were not aware of the decision to terminate the pregnancy. A few respondents elaborated on this during follow-up interviews, clarifying the guilt, anxiety and angst they felt when attending church. They accused themselves of being sinners and struggled to ask for forgiveness from God.

“You see as a married women [---] you see [---] it seems like the woman is not even ready for the marriage [---] so something is wrong with her that cannot be explained, so they [the husbands] don’t like it so easy, they see it as a sin, so there is no way I can tell about the abortion to him as I know the consequences can be bitter for me [---] when you do such thing [abortion] since you are giving away God’s blood, then you are trying to be like the Father. God gave you the child, and now you are removing it so it’s a sin because you are competing with God.” (Respondent 9)

### Choosing abortion despite risks and limited information

The main category *Choosing abortion despite being aware of the risks* is explained through three generic categories: 1) *Being aware of the risks related to abortion*, 2) *Significant others and storytelling as guides for selecting an abortion provider* and 3) *Unmet need for information concerning safe abortion methods.*

#### Being aware of the risks related to abortion

The respondents generally described abortion as risky. All of them said they were aware of the health risks of having an abortion. Death, infertility, long-time infection, weakness and loss of body weight were commonly mentioned. Death was the most emphasised consequence and was frequently repeated. Women described having an induced abortion as gambling with life and death.

“I was [very] scared [---] because I know how dangerous it is. But I was like, okay – let it be, and if I’m going to die, so be it, that is how, that is my destiny now. [---] I had now decided; it’s either death or survival. I was ready for anything.” (Respondent 5)

All women highlighted abortion as an unsafe procedure in Kenya. The respondents were fully aware of the severity of abortion complications, and their decisions were framed with this knowledge in mind. Women considered the risk of giving birth to a child to be similarly high. Going through a pregnancy was also associated with health risks (including sickness during and after pregnancy), but abortion was framed as the preferred risk. However, some respondents were anxious about the future and did not want to be blamed for their decision to terminate the pregnancy.

#### Significant others and storytelling as guides in selecting an abortion provider

Evidence-based information regarding induced abortion was limited. Common information sources about induced abortion methods and procedures were informal social networks at high school and friends who had experience of abortion. Only a few respondents had consulted professional healthcare providers. Some women stated they knew about Marie Stopes, a reliable abortion provider; however, due to high transportation fees they opted for medical abortions using Misoprostol, which was provided by chemists.

“When I was in high school we used to have some cases [of abortion] so I had that knowledge from school, so I just decided to do it on my own. [---] I also knew about other methods, but I was afraid to use the others because I had not tried to do it before.” (Respondent 9)

#### Unmet need for information concerning safe abortion methods

Respondent knowledge about safe abortion methods was low. Almost all women described induced abortions as very risky, even with the possible consequence of death. The general consensus was that no abortion is safe. Women tended to ask the local chemist about abortion drugs (Misoprostol) or quinine instead of reaching out to safe professional abortion providers due to a lack of accurate understanding of abortion legislation and safe abortion methods in Kenya.

“[Abortion is] when you take drugs [---] traditional herbals also terminate the pregnancy. Some people take juice, highly concentrated juice [---] only those once [are the abortion methods I know of].” (Respondent 6)

“I didn’t know anything. I have a friend who went through it before [---], but she passed away two weeks after the abortion [---].” (Respondent 2)

## Discussion

Similar to previous studies, this study reveals that the main reasons for induced abortion are socio-economic stresses and a lack of support from partners [28–31]. In cases where women informed their partners about the pregnancy, the principal decision maker was often the male partner who pressed for pregnancy termination indirectly by declining his financial or social responsibilities or directly by demanding the woman terminate the pregnancy. In some cases, the male partner misled the woman, overruling her decision to continue the pregnancy by arranging an unsafe abortion without her consent. A lack of financial security seemed to diminish participants’ perceptions of available options. Furthermore, as mentioned above, gender-based power relations hindered women from actualising their decisions. Previous studies from Uganda and Ghana have disclosed similar findings where women’s decision-making power regarding abortion was restricted by gender norms and power imbalances [30–32].

Similar to earlier studies, female friends or sisters were commonly referred to as important sources of information and moral support when undergoing an induced abortion. While sisters were believed to be trusted to keep knowledge about the abortion within the family, important knowledge-sharing also took place via female friends who had themselves been through an abortion. Together with sisters, these friends were regarded as trustworthy.

Regardless of relationship status, all respondents expressed concern about publicly disclosing the abortion, fearing negative remarks, the loss of social respect, isolation and divorce. Similar findings have been shown in Ethiopia, Sri Lanka and Kenya [33–35] and in further conceptualisations of abortion stigma, which entails shaming and discriminating against women and their families [36, 37]. Kumar et al. argue that abortion stigma builds on injustices and discrimination in society by depending on and appropriating existing power axes [36]. This study showed perceived stigma, referring to the perception that pregnancy termination will result in a woman being seen as inferior, to be very present in the participants’ accounts [36]. Similar to Shellenberg et al.’s arguments, the fear of judgment effectively curtailed participants’ willingness to disclose their abortion intention or experience [37]. Women handled these risks by making strategic choices regarding whom they confided in. A lack of trust and the fear of rumours confirm that other people’s opinions are highly important to sustaining a positive social life. In many cases, lying, hiding and planning to escape were preferable to telling the truth.

A direct consequence of this secrecy is that it creates a culture of silence around mistimed pregnancies and abortion. Although it may protect women from social shame, hiding one’s pregnancy and abortion makes women reluctant or scared to seek professional reproductive health information and care, which in turn makes them more vulnerable to complications, morbidities and mortality. This aligns with findings from a similar context showing that fear of stigma related to unintended pregnancy among young women, including the shame it brings to the family, as well as negative social sanctions, is a great driving force for unsafe abortion [33, 38]. The study also shows that fear of stigma delays care-seeking and consequently increases the risk of morbidity and mortality.

This study reveals that women encounter challenges in obtaining safe abortion information and services, regardless of the legal status of abortion in the country. A previous study from Kenya has similar findings [39].

### Abortion-related social stigma preventing women’s access to comprehensive SRH

Essential elements regarding abortion-related stigma found in this study should be reflected in the planning and implementation of SRH services in Kenya. Women who participated in this study repeatedly noted significant social stigma around induced abortion, which affected their decision-making regarding pregnancy termination. The abortion-related social stigma revealed in this study delayed and prevented the seeking of professional and safe PAC. Similarly, an earlier facility-based study from Kenya confirms the correlation between stigma and abortion-seeking behaviours among women seeking abortion care [40]. Young unmarried women faced both the stigma of pregnancy outside of marriage and abortion-related stigma. Previous research has shown comparable findings [41].

A recent systematic literature review critically analysed abortion stigma among healthcare providers in sub-Saharan Africa and Southeast Asia [42]. The findings demonstrate that healthcare providers have moral, social and gender-based reservations related to induced abortion. Furthermore, the study reveals that nurses and midwives often have pejorative attitudes towards women seeking abortion care and commonly reported an unwillingness to provide abortion care. As a consequence, nurses and midwives overlooked their responsibilities as caregivers and acknowledged that due to negative rapport between abortion provider and client, women seeking abortion care received inadequate care [42]. Because stigma is transmissible, it also deters healthcare providers who are prepared to provide abortion care from delivering these services. This stigmatisation enforces negative labels for the caregiver and may result in harmful professional consequences [43].

Abortion stigma is deep-rooted in government and political landscapes, organisations, communities and personal relationships [36]. The constant denial of a woman’s right to freely decide on the number and spacing of her children directly influences maternal mortality [44]. Abortion-related stigma is a barrier to safe and accessible abortion care [43, 45]. Hence, decreasing social and cultural stigma around abortion among abortion providers would potentially strengthen women’s access to quality reproductive healthcare and improve women’s health by preventing future unintended pregnancies, as well as induced abortions and related complications.

### Strengths and limitations

Despite the high prevalence of induced abortions in Kenya [19, 46], there is a shortage of qualitative research that analyses women’s reasons for obtaining induced abortions in the country.

Studies on abortion in Kenya have primarily focused on incidence, clinical outcomes and stigma [19, 39, 47, 48]. The strengths of the present study are, first, that it focusses on women who terminated their pregnancies in the recent past and thus have fresh memories of the abortion experience. Second, the interviewer was nonclinical, from outside the Kenyan healthcare system and ensured participants’ confidentiality. Third, the interviewer established a rapport with the participants, which facilitated insightful responses. Finally, six respondents agreed to a repeated interview, which provided a unique opportunity to ask follow-up questions, probe for additional information and circle back to key questions to generate richer material.

A limitation of this study is that partners and other significant family members were not included as study subjects. Partners might have given different accounts of the decision-making process. The methodological challenges in gaining access to both partners’ and parents’ accounts first and foremost relate to privacy, as partners and parents were not necessarily informed of the pregnancy and/or abortion. Ethical concerns prevented the recruitment of partners via the participants as this would require the women’s consent, which, in turn, could influence the sample. Similarly, ethical concerns prevented the inclusion of women under 18 years of age in the study and by coincidence there were no women over the age of 32 years interviewed. It could be argued that adolescent women and older women would have responded differently about decision-making preceding induced abortion. Even though research among underage women is difficult to conduct, young women are by far the most affected by severe complications due to unsafe abortions [47]. More research is therefore needed to understand underlying social attitudes towards young women who have undergone induced abortions.

Furthermore, contraceptive failure and change of pregnancy intention during pregnancy were not included in this study, which could be perceived as a limitation.

The impact of HIV status on decision-making in relation to induced abortion has been demonstrated in other studies [49, 50]. This sample included two women who reported themselves as HIV positive, which also influenced their decisions to have induced abortions (along with financial reasons). However, analysis of this relationship should be based on a larger sample.

The results from this qualitative study are the reports of PAC-seeking women in Kenya during in-depth interviews.

## Conclusions

A lack of financial independence, a lack of social support, deviance from family expectations and gender-based norms influenced abortion decision-making among women with unwanted pregnancies. Strategic choices regarding whom to confide in were employed as protection against abortion stigma. This, however, contributed to a culture of silence around abortion and mistimed pregnancy. Silence and stigma act as driving forces for unsafe abortions and put women in situations where an unsafe abortion can occur without their consent. Unwanted pregnancies can also be stressful for men, and interventions targeting unsafe abortions must take both sexes into consideration and address the problem of forced abortions as a reproductive health issue.

## Abbreviations

HIV: Human Immunodeficiency Virus
IDI: In-depth interview
JOOTRH: Jaramogi Oginga Odinga Teaching and Referral Hospital
KDH: Kisumu East District Hospital
KMET: Kisumu Medical and Education Trust
MMR: Maternal mortality ratio
PAC: Post-abortion care
SRH: Sexual and reproductive health
SRHR: Sexual and reproductive health and rights
SSA: Sub-Saharan Africa
WHO: World Health Organization
